# Minimum standards for training in colorectal endoscopic mucosal resection among advanced endoscopy trainees

**DOI:** 10.1055/a-2683-9906

**Published:** 2025-09-09

**Authors:** Dennis Yang, Ernesto Robalino Gonzaga, Muhammad Khalid Hasan, Arvind Julius Trindade, Mark Radlinski, Rebecca A Burbridge, Jeffrey Mosko, Pushpak Taunk, Salmaan Jawaid, Mohamed O. Othman, David L Diehl, Harshit S. Khara, Quin Liu, Srinivas Gaddam, Harry Aslanian, Shailendra S Chauhan, Amrita Sethi, John Poneros, Jason Samarasena, Ali M Ahmed, Uzma D. Siddiqui, Dennis Chen, Moamen Gabr, Andrew Y. Wang, C-EMR STAT Working Group

**Affiliations:** 1 440172 Center for Interventional Endoscopy, AdventHealth Orlando, Orlando, United States; 2 440172 Division of Gastroenterology, AdventHealth Orlando, Orlando, United States; 3 25049 Medicine, Division of Gastroenterology, Long Island Jewish Medical Center, New Hyde Park, United States; 4 2358 Division of Gastroenterology, University of Virginia, Charlottesville, United States; 5 609772 Department of Gastroenterology & Hepatology, Duke University Medical Center, Durham, United States; 6 10071 Gastroenterology, St Michael's Hospital, Toronto, Canada; 7 7831 Division of Digestive Disease and Nutrition, Department of Internal Medicine, University of South Florida, Tampa, United States; 8 3989 Division of Gastroenterology and Hepatology, Baylor College of Medicine, Houston, United States; 9 21599 Department of Gastroenterology and Nutrition, Geisinger Medical Center, Danville, United States; 10 22494 Digestive Disease Center, Cedars-Sinai Medical Center, Los Angeles, United States; 11 22494 Medicine, Cedars-Sinai Medical Center, Los Angeles, United States; 12 12228 Division of Gastroenterology, Yale School of Medicine, New Haven, United States; 13 2351 Gastroenterology and Hepatology, Atrium Health, Charlotte, United States; 14 21611 Gastroenterology and Hepatology, Columbia University Medical Center, New York, United States; 15 21611 Digestive and Liver Diseases, New York-Presbyterian/Columbia University Irving Medical Center, New York, United States; 16 8788 Gastroenterology, University of California Irvine, Irvine, United States; 17 14372 Gastroenterology & Hepatology, UAB Health System, Birmingham, United States; 18 2462 Gastroenterology, The University of Chicago, Chicago, United States; 19 21727 Center for Endoscopic Research and Therapeutics, The University of Chicago Medicine, Chicago, United States; 20 1859 Center for Advanced Endoscopy, Beth Israel Deaconess Medical Center, Boston, United States; 21 2358 Division of Gastroenterology and Hepatology, University of Virginia, Charlottesville, United States

**Keywords:** Endoscopy Lower GI Tract, Polyps / adenomas / ..., Endoscopic resection (polypectomy, ESD, EMRc, ...), Quality and logistical aspects, Training

## Abstract

**Background and study aims:**

Data on colorectal endoscopic mucosal resection (C-EMR) training during advanced endoscopy fellowship remain limited. We aimed to determine the number of procedures required by an “average” advanced endoscopy trainee (AET) to achieve competence in cognitive and technical C-EMR skills.

**Methods:**

AETs from advanced endoscopy training programs (AETPs) were graded on every C-EMR using a standardized assessment tool. Cumulative sum (CUSUM) analysis was used to generate individual and aggregate learning curves to estimate the minimum number of cases required to achieve competence for overall, technical, and cognitive components of C-EMR. AETs completed a self-assessment questionnaire on C-EMR competence at the end of their training.

**Results:**

A total of 22 AETs among 16 AETPs participated in this study. Nineteen AETs (86%) reported formal training in C-EMR with a mean number of 32 ± 22 cases prior to their AETP. In aggregate, 637 C-EMRs were performed (median of 32 per AET; interquartile range 17–45). Learning curve analyses revealed substantial variability in minimum volume of procedures needed to attain competence across different C-EMR skills (range: 19–39). A minimum of 19 cases were required to achieve overall competence using the global assessment score. All AETs reported feeling comfortable performing C-EMR independently at the end of AETP, yet only three (14%) achieved competence in their overall performance.

**Conclusions:**

The relatively low number of C-EMRs performed by many AETs may be insufficient to achieve competence. The estimated thresholds for an average AET to achieve competence in C-EMR provide a framework for AETPs in determining the minimal standards for case volume exposure during training.

## Introduction

Advanced endoscopy training programs (AETPs) have evolved over the years to offer training in a myriad of procedures besides endoscopic retrograde cholangiopancreatography (ERCP) and endoscopic ultrasound (EUS). Many AETPs provide additional training in colorectal endoscopic mucosal resection (C-EMR), which is widely regarded as the preferred treatment strategy for large non-pedunculated colorectal polyps (LNPCPs) 1 2 . Although several studies have shown that C-EMR outcomes are invariably dependent on the training and experience of the endoscopist 3 4 , markers of training competency are lacking.

With the increasing focus on competency-based medical education, there has been an emphasis on establishing specific standardized milestones and metrics to guide the design, implementation, assessment, and evaluation of procedure training 5 6 . In recent years, studies evaluating threshold to achieve competence in EUS and ERCP by advanced endoscopy trainees (AETs) based on standardized assessment tools have helped AETPs establish minimal standards for case volume exposure during training 7 8 . There is currently no consensus on minimum standards for C-EMR training for AETs. Although establishing a minimum procedure volume threshold does not equate to competence for all trainees, understanding the number of procedures required to achieve competence for the “average” AET in all aspects of C-EMR helps create a working framework for those AETPs providing such training. The primary aim of this study was to evaluate learning curves and define the number of procedures required by an average AET to achieve competence in all technical and cognitive aspects of C-EMR during their fellowship.

## Methods

### Study setting and subjects

This was a prospective, multicenter, cohort study that included 16 AETPs in the United States. AETs were defined as trainees who had completed a standard gastroenterology fellowship in the United States or Canada. A total of 22 AETs from the participating centers were enrolled in the study from July 2022 to July 2023. All AETs provided informed consent. The study was approved by the Human and Research Protection Office or Institutional Review Board at each participating institution, with the Center for Interventional Endoscopy (CIE) at AdventHealth, Orlando, Florida, United States serving as the central coordinating center. All authors had access to the study data and reviewed and approved the final manuscript.

### Baseline training in C-EMR

All AETs completed a questionnaire to assess their exposure to C-EMR before their fellowship (Appendix 1). The survey included questions regarding their participation in didactics and hands-on training in C-EMR through their gastroenterology fellowship, national conferences, and courses. The AETs were provided a second survey questionnaire following completion of their training to assess their perceptions and attitudes toward C-EMR training from their AETP (Appendix 2)

### Grading of AETs using a C-EMR standardized assessment tool (STAT)

AETs were graded on every C-EMR during their fellowship to capture the total number of procedures performed through their academic year. C-EMR was defined as resection of colorectal polyps in which submucosal lifting (via needle injection or water-assisted) was performed to elevate the polyp followed by snare resection. Grading was standardized and performed by attending endoscopists at each institution. The study protocol required that grading be performed shortly after each procedure to reduce recall bias, halo, and recency effect.

We used the C-EMR standardized skills assessment tool (C-EMR STAT) previously validated in a pilot study 9 to grade C-EMR skills in a continuous fashion throughout training. This tool was created by consensus opinion and review of the literature by expert endoscopists in C-EMR with the aim of including key concepts and core skills necessary for high-quality C-EMR as per the American Society for Gastrointestinal Endoscopy (ASGE) Practice Guidelines 2 9 . The C-EMR STAT contains various cognitive and technical procedure steps (Appendix 3). The four core cognitive and technical skills of C-EMR were defined as: 1) lesion assessment based on polyp morphology; 2) lesion assessment based on pit and vascular pattern; 3) submucosal injection; and 4) snare resection. A four-point scoring system was used to grade each cognitive and technical endpoint, based on the format of previously validated endoscopic assessment tools 8 10 : 4 (superior), achieves task without instruction; 3 (advanced), achieves with minimal verbal cues; 2 (intermediate), achieves with multiple verbal cues or hands-on assistance; 1 (novice), unable to complete and requires trainer to take over. Independent grading of individual endpoints was performed. These anchors allowed for the supervising endoscopists to attach behaviors and skills to anchors and ensure reproducibility over the course of the study 8 9 10 . In addition to these grading parameters, the C-EMR STAT also included pertinent information regarding procedure time, polyp characteristics (i.e. size, morphology, location), procedure difficulty based on established criteria, adverse events (AEs), and degree of AET participation (observation only, hands-on participation with assistance, or completion of the C-EMR without hands-on assistance) (Appendix 3). Polyp complexity was then categorized according to the SMSA (Size, Morphology, Site, Access) classification system 11 .

Prior to initiation of the study, the C-EMR STAT was shared with all the supervising endoscopists, who were interventional endoscopists at their respective centers with experience in C-EMR. The systematic evaluation process with this tool was explained, discussed, and clarified by the principal investigator and CIE research team. The AETs were unblinded and had access to their EMR STAT grading following each procedure.

### Data collection

All participating sites entered data on a CIE AdventHealth instance of REDCap, a secure, online database system. Each site was provided with unique logins in order to access the centralized electronic database for entry. A combination of an application programming interface, REDCap and SAS (version 9.4; SAS Institute, Cary, North Carolina, United States) were used to generate a graphic representation of overall and individual endpoint cumulative sum (CUSUM) analysis learning curves.

### Study outcomes

The primary study outcome was to identify the number of procedures required by an average AET to achieve competence in C-EMR (composite performance) and its various cognitive and technical constituents.

### Statistical analysis

**Fig. 1:**
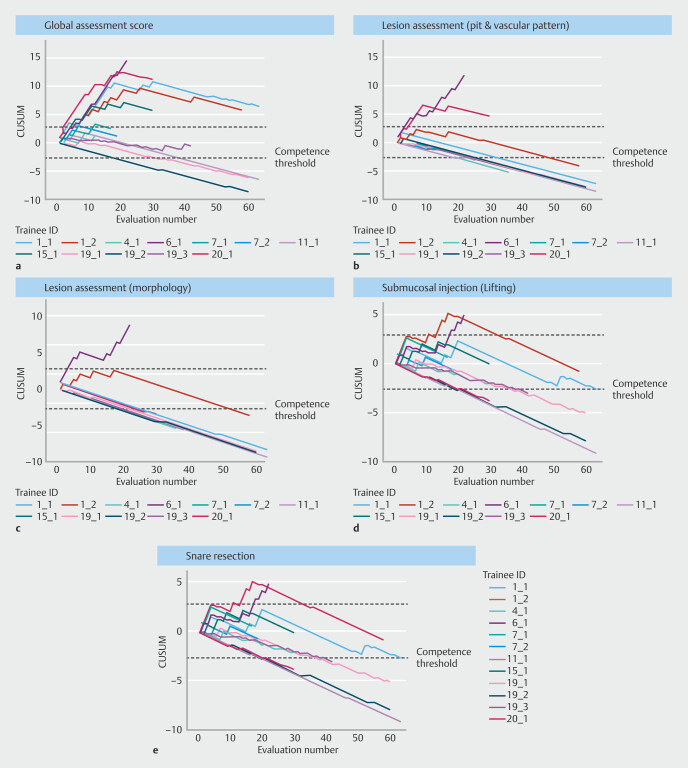
Graphical representation on CUSUM analysis applied to evaluate learning curves for core cognitive and technical skills for C-EMR. If the CUSUM curve crosses the upper decision limit from below (unacceptable threshold), the failure rates have reached preset unacceptable rates, indicating need for further training. Conversely, competence is defined when the curve crosses the lower decision limit (competence threshold) from above. If the CUSUM curve remains between the two decision limits, then ongoing observation is indicated. **a** Overall competence, based on the global score, was attained by three of 22 trainees (14%) by the end of the study. **b** Pit and vascular pattern lesion assessment competence was achieved by eight of 22 trainees (36.4%). **c** Morphology lesion assessment competence was achieved by nine of 22 trainees (40.9%). **d** Submucosal injection lifting competence was achieved by six of 22 trainees (27.3%). **e** Only three of 22 trainees (13.6%) crossed the competence threshold for snare resection.

The criterion standard for each endpoint analysis was based on the impression by the supervising endoscopist. CUSUM analysis was applied to evaluate and plot learning curves for each trainee. This statistical method permits continuous assessment of performance against a predetermined standard and has been widely applied and validated for learning in endoscopic procedures 8 9 10 12 13 . For the individual skills, we used a four-point rating system: a rating of ≥ 3 was considered a success (minimally required numeric score for competence) and a rating < 3 was considered a failure. In addition, a 10-point overall assessment score (1–3, below average; 4–6, intermediate; 7–9, advanced; 10, superior) was provided on each case. For overall performance, we used a 10-point scoring system, with success defined as above average (score of 7 to 9) or attending level (score of 10). CUSUM graphs were constructed as previously described by Bolsin and Colson 14 . A successful procedure was labeled as s and failure as 1-s. In calculating the CUSUM score, the score of s was derived from the prespecified acceptable failure rates (p0, level of inherent error if procedure is performed correctly) and unacceptable failure rates (p1, where p1-p0 represents the maximum acceptable level of human error). The CUSUM scores of both individual skills and overall performance were calculated for each AET. Learning curves were created by plotting the CUSUM scores against the index number of EMR cases by each trainee. Decision limits were calculated based on the type I (0.1) and type II (0.1) errors, p0 (10%) and p1 (20%). Fig. 1 is a graphical representation of how CUSUM analysis is applied to evaluate learning curves. If the CUSUM curve crosses the upper decision limit from below (unacceptable threshold), failure rates have reached preset unacceptable rates, indicating need for further training and the CUSUM score is reset to 0 9 14 . Conversely, competence is defined when the curve crosses the lower decision limit (competence threshold) from above. If the CUSUM curve remains between the two decision limits, then ongoing observation is indicated.

## Results

Seventy-two AETPs were invited to participate. These AETPs were identified based on their profile on the ASGE website indicating “Lower GI EMR” as a procedure offered during training. Of these programs, 41 AETPs responded to the invitation, of whom 25 declined participation because the programs did not expect their trainees to perform > 10 C-EMRs throughout the academic year. Twenty-two AETs from 16 centers agreed to participate in the study.

### Baseline C-EMR training experience

All AETs responded to the baseline survey questionnaire. Of the 22 AETs, 20 (91%) reported having undergone formal training in colorectal polypectomy and 19 (86%) in colorectal EMR. Mean numbers of polypectomies and C-EMRs reported by the AETs prior to their advance endoscopy fellowship were 358 ± 157 and 32 ± 22, respectively.

### Polyp and procedure characteristics

Colorectal polyp and C-EMR characteristics are summarized in Table 1. In aggregate, 22 AETs from 16 AETPs performed a total of 637 C-EMRs (median per AET of 32; interquartile range 17–45) during their training. Mean lesion size was 27.8 ± 12.9 mm. The three most common polyp morphologies encountered based on the Paris classification system 15 were IIa (n = 251; 39.4%), followed by Is (n = 195; 30.6%) and IIb (n = 40; 6.3%). Nearly half of the lesions were classified as lateral spreading granular lesions (n = 308; 48.4%). Most of the polyps were in the colon proximal to the splenic flexure (n = 450; 72.5%).

Mean procedure time was 27.6 ± 19.7 minutes. There were five cases (0.8%) of post-procedure bleeding within 24 hours from C-EMR. Bleeding had ceased upon repeat colonoscopy with none of these patients requiring endoscopic intervention. A single case of perforation (0.16%) occurred, which was identified during C-EMR and successfully closed endoscopically. Post-procedure imaging with enteral contrast did not show evidence of extravasation. Nearly all cases were performed in the outpatient setting, with only six patients (0.9%) admitted postoperatively. Hospital length of stay in these patients ranged between 1 and 2 days and all were discharged without AEs.

### AET participation and procedure complexity/difficulty

Of the 637 C-EMR procedures, the AETs did not have hands-on participation in 46 (7.2%) of cases. The main reasons for lack of hands-on participation were time constraints (n = 21; 3.3%) and a procedure deemed to be of high complexity/difficulty (n = 22; 3.5%) not suitable for AET participation based on supervising endoscopist discretion. The factors associated with increased procedure complexity/difficulty are shown in Table 1 .

**Table:** **Table 1** Polyp and procedure characteristics of colorectal EMR performed by advanced endoscopy trainees during the study period (N = 637).

**Polyp characteristics**	**Value**
Polyp size, mean (SD), mm	27.8 (12.9)
Polyp location, n (%)	
Appendiceal orifice	14 (2.2)
Ileocecal valve	26 (4.1)
Cecum	119 (18.7)
Ascending colon	175 (27.5)
Hepatic flexure	64 (10.0)
Transverse colon	78 (12.2)
Splenic flexure	8 (1.3)
Descending colon	31 (4.9)
Sigmoid colon	54 (8.5)
Rectum	52 (8.2)
Paris classification, n (%)	
Ip	40 (6.3)
Is	195 (30.6)
IIa	251 (39.4)
IIb	59 (9.3)
IIa+IIc	31 (4.9)
IIa+Is	47 (7.4)
III	4 (0.6)
Other	10 (1.6)
Polyp morphology, n (%)	
Lateral spreading, granular	308 (47.1)
Lateral spreading, non-granular	76 (11.9)
Lateral spreading, mixed	50 (7.8)
Not applicable	203 (31.9)
NICE (NBI International Colorectal Endoscopic) classification, n (%)	
Type 1	156 (24.5)
Type 2	391 (61.4)
Type 3	8 (1.3)
Kudo Pit Pattern classification, n (%)	
Type I	72 (11.3)
Type II	64 (10.0)
Type III-L	129 (20.3)
Type III-S	117 (18.4)
Type IV	38 (6.0)
Type V	17 (2.7)
Procedure characteristics	
Type of submucosal injection, n (%)	
Normal saline	32 (5.0)
Viscous solution	557 (87.4)
Not applicable	48 (7.5)
Total EMR time, mean (SD), minutes	27.6 (19.7)
Adverse events, n (%)	
Bleeding	5 (0.8)
Perforation	1 (0.16)
Advanced endoscopy trainee involvement, n (%)	
Observation only	46 (7.2)
With hands-on assistance	209 (32.8)
Without hands-on assistance	382 (60)
Reasons for lack of hands-on participation by the trainee, n (%)	
Time constraints	21 (3.3)
Complex/difficult procedure not suitable for trainee	22 (3.5)
Endoscopic resection was not performed	6 (0.9)
Factors associated with increased procedural complexity/difficulty, n (%)	
Difficult access	72 (11.3)
Difficult position	220 (34.5)
Unfavorable gravity position	75 (11.8)
Prior attempted resection	52 (8.2)
Tattoo at the base of the polyp	64 (10.0)
Non-lifting sign	41 (6.4)
Polyp size ≥ 40 mm	95 (14.9)
EMR, endoscopic mucosal resection; SD, standard deviation.	

### C-EMR learning curve and competence

Data from all AETs were used to generate the CUSUM learning curves to estimate the competence threshold for the various individual cognitive and technical C-EMR endpoints. There was substantial variability in the minimum threshold of procedures needed to attain competence across different cognitive and technical C-EMR skills ( Table 2 ). The CUSUM curves for the core cognitive and technical skills for C-EMR are shown in Fig. 1. By the end of training, competence in cognitive skills was achieved by nine (40.9%) for assessment of polyp morphology and by eight (36.4%) for assessment of pit/vascular pattern. Only six (27.3%) and three (13.6%) of the AETs crossed the competence threshold for submucosal injection and endoscopic resection, respectively. Overall competence, based on the global score, was attained by three of the 22 trainees (14%) by the end of the study ( Fig. 1 ).

**Table:** **Table 2** Minimum procedure volume thresholds to attain competence across cognitive and technical C-EMR skills.

**Colorectal EMR standardized assessment tool**	**Minimum number of procedures needed to attain competence in each skill category, n**
**Cognitive skills**	
Lesion assessment (polyp morphology)	19
Lesion assessment (pit and vascular pattern)	19
Identification of features suggestive of submucosal invasion	19
Recognize muscle injury following C-EMR (safety)	19
**Technical skills**	
Adequate access and positioning of scope in relation to the lesion	19
Submucosal injection	29
Snare resection	23
Adjunct mechanical resection techniques (example: avulsion with forceps)	36
Adjunct ablative techniques (example: snare tip soft coagulation of mucosal defect margins)	25
Management of bleeding	39
Elective closure	22
**Global scale**	19
EMR, endoscopic mucosal resection.	

### AET self-assessment on completion of C-EMR training

Nineteen AETs completed the self-assessment questionnaire at the end of their training ( Table 3 ). From a cognitive skills standpoint, all AETs strongly agreed or tended to agree with the statement that they were comfortable recognizing the indication and contraindications for C-EMR and classifying polyps based on their morphology; although three trainees (14%) disagreed with the statement that they could routinely classify a lesion based on its vascular and pit pattern. Nearly all AETs agreed (18/19; 94.7%) about feeling comfortable performing all technical C-EMR skills and independently performing C-EMR at the end of their training.

**Table:** **Table 3** Survey questionnaire to AETs about their C-EMR training after completion of fellowship.

**Statement**	**Response**				
	**Strongly Agree**	**Tend to Agree**	**Neutral**	**Tend to Disagree**	**Strongly Disagree**
Comfortable with independently performing C-EMR at the endo of training	16	2	1	--	--
I am comfortable at recognizing the indications and contraindications for C-EMR	17	2	--	--	--
I can routinely classify a lesion based on its vascular and pit pattern	10	5	1	3	--
I am comfortable achieving adequate positioning for C-EMR	13	6	--	--	--
I am comfortable obtaining a submucosal lift for C-EMR	16	2	1	--	--
I am comfortable with underwater C-EMR	9	7	--	2	1
I can routinely obtain en-bloc resection for lesions < 20 mm	15	4	--	--	--
I feel comfortable using adjunct resection and ablative techniques for the removal of residual polyp	13	4	--	--	--
I am comfortable managing bleeding during C-EMR	14	4	1	--	--
I am comfortable managing perforation during C-EMR	10	7	2	--	--
C-EMR, colorectal endoscopic mucosal resection.					

## Discussion

C-EMR is the most widely accepted first-line treatment of large non-malignant colorectal polyps in clinical practice. Although most AETPs offer training in C-EMR according to the information provided to the ASGE advanced endoscopy fellowship match website, there is a lack of formal data on C-EMR training and there is no fixed mandatory curriculum and no set minimum standards. In this study of 22 AETs from 16 AETPs, we identified the minimum threshold for an average AET to achieve overall competence in C-EMR as approximately 19 cases, with significant variability in the number of C-EMRs performed by AETs among the different participating AETPs. Overall, these data may provide a framework for AETPs in determining minimum standards for case volume exposure in C-EMR during training.

The main training focus of most AETPs still revolves around ERCP and EUS. However, in recent years, many AETPs have expanded their curricula to include training in procedures across various subspecialties of interventional endoscopy, such as submucosal endoscopy, endoscopic resection, and bariatric endoscopy 16 . With this expanding portfolio, it is important for AETPs to understand how many C-EMR procedures the average AET will require during their fixed training period to achieve competency. Understanding this threshold volume has important implications. For instance, on the ASGE website, 72 AETPs indicated that “lower GI EMR” is a procedure that is being offered during training. However, it remains unclear how many of these programs actually expose and train their AETs in a substantial number of C-EMRs. Indeed, more than half of the programs indicating that they train their AETs in C-EMRs on the ASGE website ended up declining participation (25/41; 61%) in this study citing their concern that most of their trainees do not perform > 10 C-EMRs during the fixed training period. Hence, identifying the minimum threshold required to achieve competency in C-EMR by the average AET provides a benchmark for programs to determine whether this will be a procedure for which they can realistically offer training. We hope that this knowledge will be useful to AETPs committed to training AETs in C-EMR and that it will inform prospective AETs interested in learning C-EMR so that they can identify programs that offer sufficient volume in this procedure to attain competency.

Overall, our study suggests that comprehensive C-EMR training within the constraints of a standard 12-month AETP can be challenging. There was significant variability in the number of C-EMR cases by AETs (range 5 to 120), with 11 of the AETs (50%) performing < 19 procedures during training. None of the AETs with less than 19 C-EMR procedures completed were able to attain competence in any of the cognitive and technical skills components or on their global assessment. Notably, the number of C-EMR procedures required to cross the competency threshold for management of procedure-related AEs (i.e. bleeding, perforation) was even higher. Patient safety should be a key factor when determining competency. Hence, we strongly believe that formal assessment of a trainee’s ability to manage procedure-related complications should be an important component when establishing their readiness for independent practice. Although it is clear that trainees acquire endoscopic skills at variable rates and volume thresholds do not necessarily ensure competence, our findings further emphasize the value of establishing minimum standards with regard to procedure volume. It should be emphasized that irrespective of the number of C-EMRs the trainees performed during the study period, all AETs had already completed a general gastroenterology fellowship, which included exposure and/or hands-on training on C-EMR. This in turn raises the important question about whether further emphasis and structured assessment of C-EMR training should be initiated at earlier stages during general gastroenterology fellowship. Our study demonstrated a discrepancy between AET self-assessment and their performance based on their evaluations. These findings are consistent with those from our pilot study 9 . At completion of their training, nearly all AETs indicated feeling comfortable with cognitive and technical aspects of C-EMR; nearly all also reported feeling comfortable with performing C-EMR in independent practice. Yet, data based on CUSUM analysis demonstrated that most trainees did not meet competence thresholds. Our results have important implications. For one, they suggest that trainee self-assessment should not replace formal assessment by trainers and underscore the importance of using standardized procedure-specific structured assessment tools. Despite being evaluated by trainers on each C-EMR using the C-EMR STAT as part of this study, it remains unclear whether the AETs received regular input on their performance, which may have accounted for the discrepancy. Providing structured feedback to trainees at regular intervals may potentially unmask unrecognized areas for improvement and permit corrective measures throughout their fellowship 17 18 . Whether periodic feedback using C-EMR STAT scores may translate into improved training outcomes based on objective measures needs to be addressed in future studies.

This study is not without limitations. First, this study did not include all AETPs in the United States, thereby limiting generalizability of our results. Second, there is a potential for selection bias among AETs and their programs who opted to participate in the study. We recognize that the subjective assessment of the trainer was regarded as the criterion standard. This is a common limitation of any study evaluating learning curves and competency. Similarly, interobserver and intraobserver variability among trainers, differences in experience and training styles, and variations in C-EMR techniques were also not accounted for in this study. We attempted to address these issues by using a previously established standardized assessment tool with well-defined anchors for all specific endpoints that was reviewed by all trainers prior to study enrollment. However, variance across different trainers and institutions may have impacted our results. Lastly, the relatively low and highly variable number of C-EMRs performed by AETs limited meaningful individual learning curve analyses and competence assessment among those with very few procedures and when evaluating certain C-EMR-related endpoints. Nonetheless, rather than a limitation of study design, the low volume numbers may simply be a reflection of the current status of C-EMR training among AETPs.

## Conclusions

In conclusion, this prospective multicenter study provided data on minimum thresholds for an average AET to achieve competence in various cognitive and technical aspects of C-EMR. The relatively low number of C-EMRs performed by many AETs may be insufficient to achieve competence, despite their prior exposure to this procedure during general gastroenterology fellowship and reporting feeling comfortable performing C-EMR independently in clinical practice. Our data provide a framework for AETPs to adjust their educational curricula in response to the increasing number and complexity of advanced endoscopic techniques to which trainees are exposed during their training.

## Supplementary Material

Supplementary Material Zusatzmaterial

## References

[1] Meulen LWT van der Zander QEW Bogie RMM Evaluation of polypectomy quality indicators of large nonpedunculated colorectal polyps in a nonexpert, bowel cancer screening cohort Gastrointest Endosc 2021 94 1085 1095 10.1016/j.gie.2021.06.008 34139253

[2] Kaltenbach T Anderson JC Burke CA Endoscopic removal of colorectal lesions – Recommendations by the US Multi-Society Task Force on Colorectal Cancer Gastrointest Endosc 2020 91 486 519 10.14309/ajg.0000000000000555 32067745

[3] Bhurwal A Bartel MJ Heckman MG Endoscopic mucosal resection: learning curve for large nonpolypoid colorectal neoplasia Gastrointest Endosc 2016 84 959 968 10.1016/j.gie.2016.04.020 27109458

[4] Rajendaran A Pannick S Thomas-Gibson S Systematic literature review of learning curves for colorectal polyp resection techniques in lower gastrointestinal endoscopy Colorectal Dis 2020 22 1085 1100 31925890 10.1111/codi.14960

[5] Wani S Keswani RN Petersen B Training in EUS and ERCP: standardizing methods to assess competence Gastrointest Endosc 2018 87 1371 1382 10.1016/j.gie.2018.02.009 29709305

[6] Wani S Keswani RN Han S Competence in endoscopic ultrasound and endoscopic retrograde cholangiopancreatography, from training through independent practice Gastroenterology 2018 155 1483 1494 30056094 10.1053/j.gastro.2018.07.024 PMC6504935

[7] Wani S Han S Simon V Setting minimum standards for training in EUS and ERCP: results from a prospective multicenter study evaluating learning curves and competence among advanced endoscopy trainees Gastrointest Endosc 2019 89 1160 1168 e9 30738985 10.1016/j.gie.2019.01.030 PMC6527477

[8] Wani S Keswani R Hall M A prospective multicenter study evaluating learning curves and competence in endoscopic ultrasound and endoscopic retrograde cholangiopancreatography among advanced endoscopy trainees: The Rapid Assessment of Trainee Endoscopy Skills Study Clin Gastroenterol Hepatol 2017 15 1758 1767 28625816 10.1016/j.cgh.2017.06.012 PMC7042954

[9] Yang D Perbtani YB Wang Y Evaluating learning curves and competence in colorectal EMR among advanced endoscopy fellows: a pilot multicenter prospective trial using cumulative sum analysis Gastrointest Endosc 2021 93 682 690 e4 32961243 10.1016/j.gie.2020.09.023

[10] Wani S Hall M Wang AY Variation in learning curves and competence for ERCP among advanced endoscopy trainees by using cumulative sum analysis Gastrointest Endosc 2016 83 711 719 26515957 10.1016/j.gie.2015.10.022

[11] Gupta S Miskovic D Bhandari P A novel method for determining the difficulty of colonoscopic polypectomy Frontline Gastroenterol 2013 4 244 248 10.1136/flgastro-2013-100331 28839733 PMC5369843

[12] Han S Obuch JC Keswani RN Effect of individualized feedback on learning curves in EGD and colonoscopy: a cluster randomized controlled trial Gastrointest Endosc 2020 91 882 893 10.1016/j.gie.2019.10.032 31715173

[13] Lipman G Markar S Gupta A Learning curves and the influence of procedural volume for the treatment of dysplastic Barrett’s Gastrointest Endosc 2020 92 543 550 e1 10.1016/j.gie.2020.02.041 32145288

[14] Bolsin S Colson M The use of the cusum technique in the assessment of trainee competence in new procedures Int J Qual Health Care 2000 12 433 438 10.1093/intqhc/12.5.433 11079224

[15] The Paris endoscopic classification of superficial neoplastic lesions: esophagus, stomach, and colon: November 30 to December 1, 2002. Gastrointest Endosc 2003; 58: S3-S43 10.1016/s0016-5107(03)02159-x 14652541

[16] Yang D Wagh MS Draganov PV The status of training in new technologies in advanced endoscopy: from defining competence to credentialing and privileging Gastrointest Endosc 2020 92 1016 1025 10.1016/j.gie.2020.05.047 32504699 PMC7267783

[17] Dilly CK Sewell JL How to give feedback during endoscopy training Gastroenterology 2017 153 632 636 10.1053/j.gastro.2017.07.023 28757268

[18] Thurasingam AI MacDonald J Shaw IS Insights into endoscopy training: a qualitative study of learning experience Med Teach 2006 27 453 459 10.1080/01421590600825417 16973460

