# Supplementary material for: Minimum standards for training in colorectal endoscopic mucosal resection among advanced endoscopy trainees
Source: Endosc Int Open. 2025 Sep 9;13:a26839906. doi: 10.1055/a-2683-9906 (PMC12445247; doi:10.1055/a-2683-9906)
Supplement: Supplementary file 1 — Supplementary Material [file 10-1055-a-2683-9906_26846488.pdf]

APPENDIX 1.
Study Center ID: _________ Study Participant ID: _________

ADVANCED ENDOSCOPY TRAINEE BASELINE QUESTIONNAIRE

1. How many colonoscopies did you perform during GI fellowship: _____________
2. Did you undergo formal training on the **cognitive aspects** (i.e. indications/contraindications, benefits, risks, limitations of the procedure, components of pre-endoscopic evaluation and post-procedural care) of any of the following techniques:

   a. Colorectal polypectomy: ☐ Yes ☐ No
   b. Colorectal endoscopic mucosal resection (EMR): ☐ Yes ☐ No
   c. Endoscopic submucosal dissection (ESD): ☐ Yes ☐ No

If yes, please specify (mark all that apply)

	GI fellowship	Regional/National Meetings/Conferences	Endoscopy Courses	Self-didactic learning
Colorectal Polypectomy				
Colorectal EMR				
ESD				

3. Did you observe any of the following procedures prior to the initiation of your current training?

   a. EMR ☐ Yes ☐ No If yes, how many: ______
   b. ESD ☐ Yes ☐ No If yes, how many: ______

4. If yes to question #2, please specify (mark all that apply):

	Live cases at my institution	Live cases at national GI meetings/conferences	Live cases at EMR/ESD courses	Videos online
Polypectomy				
Colorectal EMR				
ESD				

5. Please write down in each column the total number of cases (hands-on experience) that you have performed for each listed procedure prior to the current study (if applicable):

	Explant Model Cases	Live Animal Cases	Human Cases
Polypectomy			
Colorectal EMR			
ESD			

APPENDIX 2.

ADVANCED ENDOSCOPY TRAINEE POST-TRAINING QUESTIONNAIRE

1. How many colorectal endoscopic mucosal resection (EMR) were performed at your institution during the study period? ___________
2. How many of these colorectal EMR did you observe during your training? ___________
3. How many colorectal EMR did you participate (hands-on) during your training?

   ____

4. How many colorectal EMR did you complete on your own (without hands on assistance from your supervising endoscopist)? ____________
5. Please answer the following questions for each one of the following procedures by marking one of the corresponding boxes:

**Colorectal Endoscopic Mucosal Resection (COLORECTAL EMR)**	Strongly Agree	Tend to Agree	Neutral	Tend to Disagree	Strongly Disagree
I feel comfortable with independently performing colorectal EMR at the end of my advanced endoscopy training					
I am comfortable at recognizing the indications and contraindications for colorectal EMR					
I can routinely classify a lesion based on its morphology (i.e. Paris classification)					
I can routinely classify a lesion based on its vascular and pit pattern (i.e. NICE, Kudo classification)					
I am comfortable achieving adequate positioning for colorectal EMR					
I am comfortable obtaining a submucosal lift for colorectal EMR					
I am comfortable with underwater colorectal EMR					
I can routinely obtain en-bloc resection for lesions < 20 mm					

Endosc Int Open | DOI 10.1055/a-2683-9906 | © The Author(s).

I feel comfortable using adjunct resection and ablative techniques for the removal of residual polyp					
I am comfortable managing bleeding during colorectal EMR					
I am comfortable managing perforation during colorectal EMR					

APPENDIX 3.

EMR Standardized Assessment Tool (EMR-STAT)

Colorectal EMR: COGNITIVE SKILLS

1 (novice): unable to complete
2 (intermediate): achieves with multiple verbal cues or hands-on assistance
3 (advanced): achieves with minimal verbal cues
4 (superior): achieves without instruction
N/A: not applicable (the skill was not performed by neither the trainee or supervising endoscopist during the procedure)

**LESION ASSESSMENT (MORPHOLOGY):** Examines the lesion and is able to classify the polyp based on the Paris Classification	1	2	3	4	N/A
**LESION ASSESSMENT (VASCULAR AND PIT PATTERN):** Examines the lesion (including margins) using white light, digital and/or dye chromoendoscopy, near focus imaging to interpret mucosal, pit and vascular patterns and is able to classify the lesion based the NICE and/or Kudo classification system	1	2	3	4	N/A
**IDENTIFICATION OF SUBMUCOSAL INVASION:** Identifies features (ulceration, lesion depression, irregularity, absent pit/vascular pattern) that may suggest submucosal invasion when present	1	2	3	4	N/A
**SAFETY** Identifies injury to the muscularis propria (deep mural injury) during resection	1	2	3	4	N/A

Colorectal EMR: TECHNICAL SKILLS

1 (novice): unable to complete
2 (intermediate): achieves with multiple verbal cues or hands-on assistance
3 (advanced): achieves with minimal verbal cues
4 (superior): achieves without instruction
N/A: not applicable (the skill was not performed by either the trainee or supervising endoscopist during the procedure)

**ACCESS/POSITIONING:** Achieves stable endoscopic position/readjustment for EMR	1	2	3	4	N/A
**SUBMUCOSAL LIFTING:** Achieves adequate submucosal lifting (adequate separation of lesion from underlying muscle layer for safe resection)	1	2	3	4	N/A
**RESECTION:** For lesions ≤15 to 20 mm: Achieves en-bloc resection For lesions > 20 mm: Achieves en-bloc resection when feasible OR performs effective sequential piecemeal resection (defined as systematic use of snare for resection resulting in clean base with no residual islands and clear outer margins)	1	2	3	4	N/A
**ADJUNCTIVE MECHANICAL RESECTION TECHNIQUES:** Appropriate use of biopsy forceps (cold or hot) to remove residual tissue (accurate targeting with tenting followed by pulling without incurring in deep mural injury)	1	2	3	4	N/A
**ADJUNCTIVE ABLATIVE TECHNIQUES:** Effective ablates the outer resection margins using APC or the tip of the snare (can continuously ablate the margins without leaving "skip" untreated margins)	1	2	3	4	N/A
**MANAGEMENT OF BLEEDING:** Effectively intraprocedural hemostasis (cessation of bleeding).	1	2	3	4	N/A
**MANAGEMENT OF PERFORATION:** Achieves endoscopic closure (successful apposition without visible defects) of deep mural injury	1	2	3	4	N/A
**ELECTIVE CLOSURE:** Achieves elective closure of resection bed (clips, sutures, etc)	1	2	3	4	N/A

GENERAL ASSESSMENT (SUBJECTIVE):

**1**	**2**	**3**	**4**	**5**	**6**	**7**	**8**	**9**	**10**
Novice: unable to complete EMR			Intermediate: achieves EMR with multiple verbal cues and/or hands-on assistance			Advanced: Achieves EMR with minimal verbal cues			Superior: Achieves EMR without instruction
